# Down-Regulation of ZnT8 Expression in INS-1 Rat Pancreatic Beta Cells Reduces Insulin Content and Glucose-Inducible Insulin Secretion

**DOI:** 10.1371/journal.pone.0005679

**Published:** 2009-05-25

**Authors:** Yi Fu, Wei Tian, Emily B. Pratt, Lisa B. Dirling, Show-Ling Shyng, Charles K. Meshul, David M. Cohen

**Affiliations:** 1 Division of Nephrology & Hypertension, Departments of Medicine, Cell and Developmental Biology, Oregon Health & Science University, Portland, Oregon, United States of America; 2 Department of Physiology and Pharmacology, Oregon Health & Science University, Portland, Oregon, United States of America; 3 Department of Behavioral Neuroscience, Oregon Health & Science University, Portland, Oregon, United States of America; 4 Department of Pathology, Oregon Health & Science University, Portland, Oregon, United States of America; 5 Center for Research in Occupational and Environmental Toxicity, Oregon Health & Science University, Portland, Oregon, United States of America; 6 The Research Service, Portland V.A. Medical Center, Portland, Oregon, United States of America; University of Bremen, Germany

## Abstract

The *SLC30A8* gene codes for a pancreatic beta-cell-expressed zinc transporter, ZnT8. A polymorphism in the *SLC30A8* gene is associated with susceptibility to type 2 diabetes, although the molecular mechanism through which this phenotype is manifest is incompletely understood. Such polymorphisms may exert their effect via impacting expression level of the gene product. We used an shRNA-mediated approach to reproducibly downregulate ZnT8 mRNA expression by >90% in the INS-1 pancreatic beta cell line. The ZnT8-downregulated cells exhibited diminished uptake of exogenous zinc, as determined using the zinc-sensitive reporter dye, zinquin. ZnT8-downregulated cells showed reduced insulin content and decreased insulin secretion (expressed as percent of total insulin content) in response to hyperglycemic stimulus, as determined by insulin immunoassay. ZnT8-depleted cells also showed fewer dense-core vesicles via electron microscopy. These data indicate that reduced ZnT8 expression in cultured pancreatic beta cells gives rise to a reduced insulin response to hyperglycemia. In addition, although we provide no direct evidence, these data suggest that an *SLC30A8* expression-level polymorphism could affect insulin secretion and the glycemic response *in vivo*.

## Introduction

An understanding of the relationship between zinc and diabetes has evolved over decades. In insulin-containing vesicles of the pancreatic beta cells, crystalline insulin is packaged with zinc (reviewed in: [1]); in commercial preparations, insulin is crystallized with zinc to slow absorption and onset of action following subcutaneous administration. Perturbations in zinc homeostasis disrupt carbohydrate metabolism; however, the inverse relationship is also true: diabetes and hyperglycemia alter zinc balance, promoting hypozincemia and hyperzincuria (reviewed in: [2]).

Interest in the role of zinc in the pathogenesis of diabetes was re-ignited with the discovery, in five independent human genome-wide association studies, of an association between type 2 diabetes and a genetic polymorphism in the *SLC30A8* gene [3]–[7]. This gene codes for a newly described zinc transport protein, ZnT8 [8]; the minor allele of the single-nucleotide polymorphism (rs13266634) introduces a non-conservative substitution (i.e., Arg-to-Trp) in amino acid 325. This polymorphism was subsequently shown to be associated with the presence of altered glucose homeostasis, pancreatic beta-cell dysfunction, or overt type 2 diabetes in many [9]–[14] but not all [15], [16] study populations. In addition to its putative role in type 2 diabetes, ZnT8 may also serve as an autoantigen in type 1 diabetes [17].

Chimienti and co-workers originally described ZnT8 (the product of the *SLC30A8* gene) as a pancreatic islet-expressed [8], [18] protein belonging to the ZnT family of intracellular zinc transport proteins. ZnT8 co-localized with insulin-containing secretory vesicles in cultured rat INS-1 cells [8], a pancreatic beta cell line derived from a rat insulinoma [19], and in human islet cells [18]. HeLa cells heterologously expressing the transporter exhibited increased zinc uptake in the presence of excess extracellular zinc [8], as measured by the cell-permeant fluorescent zinc indicator dye, zinquin [20].

We show here that shRNA-mediated downregulation of ZnT8 in rat INS-1 insulinoma cells reduces uptake of exogenous zinc, as evidenced by zinquin fluorescence. The ZnT8-downregulated cells showed reduced insulin content and decreased insulin secretion in response to hyperglycemic stimulus, as determined by insulin immunoassay. ZnT8-depleted cells also showed fewer dense-core vesicles via electron microscopy. Many genetic polymorphisms influence phenotype by altering the level of expression of their respective proteins; some authorities feel that these expression-level polymorphisms are more significant – on a population-wide basis – than are polymorphisms that directly impact protein structure or function [21]–[29]. Although it is unclear what role – if any – the diabetes-associated *SLC30A8* polymorphism plays in aberrant glucose homeostasis, our data suggest that a polymorphism impacting only ZnT8 expression level might be sufficient to alter beta-cell function and glucose metabolism *in vivo*.

## Methods

### Downregulation of ZnT8 in INS-1 cells using shRNA stable transfection

Parental INS-1 cells were maintained in RPMI medium (which includes no added zinc) supplemented with 10% fetal bovine serum, sodium pyruvate (1 mM), HEPES (10 mM), and beta-mercaptoethanol (50 uM). Cells were stably transfected with pRS vector (Origene # TR20003; cell line Vec-shRNA), pRS-shGFP (Origene # TR30003; encoding non-effective 29-mer shGFP cassette as a negative control; cell line GFP-shRNA), or with a combination of specific ZnT8-directed 29-mer oligos (ZnT8-shRNA; see Table 1). These 29-mers were incorporated in the forward and in the reverse orientation, separated by a TCAAGAG loop, in the vector pRS shRNA by the manufacturer to generate four pRS-based gene-specific rat ZnT8 shRNA expression vectors (HuSH; Origene). All were independently tested for their ability to down-regulate ZnT8 expression. The two most effective vectors (ZnT8-3 and ZnT8-4) were then transfected in combination under puromycin selection (2 µg/ml) to generate a pooled stable cell line, and were compared to INS-1 cells stably transfected (in parallel) with empty shRNA vector or with GFP-directed shRNA. This procedure was repeated two more times such that three complete and independent sets of Vec-shRNA and ZnT8-shRNA stable transfectants were produced and analyzed. The phenotype of these cell lines remained stable for months in continuous culture; nonetheless, frozen stocks were routinely thawed to ensure consistency throughout. Grossly, morphology (via light microscopy) and growth properties of the disparate cell lines were indistinguishable. We were unable to demonstrate ZnT8 expression at the protein level using a commercially available reagent (RZ8 anti-rat ZnT8 polyclonal antibody; Mellitech, Grenoble, France). Although this reagent was used successfully by other investigators (e.g., [8], [18]), in our hands, specific signal corresponding to the appropriate molecular mass could not be discerned via immunoblotting in wild-type INS-1 cells or in protein lysates prepared from rat pancreatic tissue (data not shown).

**Table 1:** Effect of ZnT8-directed and irrelevant shRNA constructs upon ZnT8 expression in INS-1 cells.

Name	29-mer Target Sequence	29-mer ID	ZnT8/Vector
vector	N/A	TR20003	1.00
GFP		TR30003	1.04
ZnT8-1	ATGAGTCCAAGTGATCATCCAAGAAGACC	TI100047	1.87
ZnT8-2	CTGCTACCATGGAGTTTCTTGAGAGGACT	TI100048	1.20
ZnT8-3	TGTGAGCGCCTCTTGTATCCTGATTACCA	TI100049	0.63
ZnT8-4	AGTGAACCAAGTGATTCTCTCTGTTCATG	TI100050	0.54

### Real-time PCR

Total cellular RNA was isolated from stable transfectants using TriZol reagent (Invitrogen) in accordance with the manufacturer's directions. Total RNA (5 µg) was used to generate cDNA with the SuperScript III First-Strand Synthesis System (Invitrogen); product (3 µl) was amplified with TaqMan Universal PCR Master Mix (Applied Biosystems) on a StepOne Plus platform (Applied Biosystems). Rat-specific probe sets were obtained from ABI as follows: ZnT8, XM_235269-sp13 (custom); ZnT4, Rn00597094_m1 (ready-made); ZnT5, Rn01493874_m1 (custom); calcium channel, voltage-dependent, L-type, alpha 1C subunit, Rn00709287_m1 (CACNA1C, ready-made); and calcium channel, voltage-dependent, L-type, alpha 1D subunit, Rn00568820_m1 (CACNA1D, ready-made). Comparisons were made using the ΔΔC_t_ method [30] where a probe set directed against rat 18S RNA and run in parallel served as an internal control for each RNA. Assays were not multiplexed; all probe sets were FAM-based. Although C_t_ can not be directly compared across multiple probe sets (in contrast to data utilizing a single probe set), the average C_t_ for the various probe sets (in Vec-shRNA transfectants) was as follows: ZnT8, 21.7; ZnT4, 24.5; ZnT5, 25.2; CACNA1C, 26.3; CACNA1D, 31.8; 18S, 8.0 (data set used for figures).

### Zinquin fluorescence and image analysis

For zinquin staining, INS-1 cells were passaged in complete medium with 10% Chelex-treated FBS (e.g., [31]) on day 0. On day 1, cells were treated for 3 h with control (zinc-free) medium, or medium supplemented with 75 µM ZnCl_2_ or NiCl_2_. Cells were then washed×3, incubated for 30 min in HBSS in the presence of zinquin (5 µM, unless otherwise indicated), and washed×3 with HBSS [8]. The plate was then read immediately in a FlexStationII at emission wavelength 490 nm in the presence of excitation at 370 nm with an auto-cutoff set at 475 nm and photomultiplier (PMT) gain set to HIGH. Raw fluorimetric data (e.g., as in figures) are the averaged instrument readings for the indicated number of replicate wells. Owing to their highly reproducible nature, these data were not normalized. Background was taken to be the fluorimetric reading in the absence of zinquin indicator dye (e.g., the white bars in figures; ∼60,000 units). The FlexStation II instrument was programmed to read each microplate well in six separate regions and average them prior to reporting a value for each well. These parameters and photomultiplier gain were kept constant throughout all experiments.

For live-cell imaging, cells were grown in multiwell plates, and treated with Zn^2+^ or Ni^2+^ and then loaded with zinquin as described above. After the final wash, cells were returned to HBSS and observed under epifluorescence conditions in a Leica DMIRB inverted microscope with a Xenon-based Lambda LS light source, with emission monitored at 510 nm in the presence of excitation at 380 nm. Monochrome images were recorded with a Hamamatsu ORCA-ER charge-coupled device digital camera using OpenLab software (Improvision), and converted to .tiff files for analysis. For direct comparison between conditions (e.g., zinc-treated *vs.* nickel-treated), all imaging parameters (i.e., gain, binning, and exposure duration) were kept constant. Image analysis was done strictly in parallel: phase contrast images 2A and 2C were combined into a single .tiff file prior to image processing; and epifluorescence images B and D were combined into a single .tiff file prior to image processing. In each of these combined image files, minor adjustment in brightness were made using PhotoShop. For the combined epifluorescence image represented by panels B and D, contrast was enhanced such that input maximum per pixel was reduced from 255 to 175 for clarity and to more faithfully reproduce on the printed page the image viewed through the eyepiece.

### Insulin content and secretion

INS-1 cell insulin secretion and insulin content were measured via enzyme-linked immunosorbent assay directed against rat insulin (ALPCO Diagnostics; Insulin (Rat) High Range EIA). INS-1 cells were passaged on day 0 at a density of 1×10^6^ cells/well into 6-well plates in complete medium (containing 11.1 mM glucose). On day 1, medium was changed to fresh medium with 3 mM glucose×∼16 h (i.e., overnight). On day 2, cells were washed once with HBSS (NaCl 114; NaHCO_3_ 25.5; KCl 4.7; MgCl_2_ 1; KH_2_PO_4_ 1.2; MgSO_4_ 1.16; HEPES 20; CaCl_2_ 2.5; [all in mM]) supplemented with 3 mM glucose and 0.2% freshly-prepared BSA (“HBSS Plus”). Cells were then incubated for 2 h in fresh HBSS Plus, supplemented with glucose (to a final concentration of 6 or 12 mM, as indicated) or KCl (to a final concentration of 30 mM). After the 2-h incubation, supernatant was collected and stored at −20 C overnight, centrifuged to remove any debris, and then used for the secreted insulin assay. Acidified ethanol (1 ml; 75% ethanol plus 1.5% HCl) was added to monolayers in wells and cells were incubated overnight at −20 C. On day 3, these acidified ethanol extracts were collected, centrifuged to remove any debris, and used for the intracellular insulin assay. Total insulin (in ng/ml) was calculated as secreted insulin+intracellular insulin, and percent secreted insulin was defined as (100*(secreted insulin/total insulin)). We used this more specific strategy, rather than normalizing to total cellular protein, because the insulin content differed among the cell lines; we reasoned that this expression of secreted insulin as a fraction of total cellular insulin content, as other groups have done (e.g., [32], [33]) would be the most conservative approach in this instance. Because both intracellular and secreted insulin would need to be normalized to total cellular protein, were this alternative approach to be adopted (i.e., normalization to total cellular protein), the quotient would be identical to the numbers we report. Of note, total protein content per well was equivalent in the VEC-shRNA and ZnT8-shRNA cell lines (see Results). Were we to have expressed insulin secretion on a “per cell” or “per mg total protein” basis, then the consequence of ZnT8 downregulation vis-à-vis insulin secretion would have been more pronounced; however, our normalization to total insulin content (rather than protein content) permitted the VEC-shRNA and ZnT8-shRNA bars to be roughly equivalent under control conditions (3 mM glucose) in figures. The immunoassay was then carried out in accordance with the manufacturer's directions (ALPCO 80-INSRTH-E01). Absorbance was read at 450 nm in a microtiter plate reader (Molecular Devices). Where indicated, protein concentration was measured using the DC Protein Assay (BioRad) according to the manufacturer's directions (Standard Assay Protocol).

### Electron microscopy

For electron microscopy, cells were released from the plate with trypsin, quenched with 10% FBS-containing medium, and gently pelleted in a microcentrifuge tube at 1000 *g*. The supernatant was removed and replaced with fixative (2.5% glutaraldehyde/0.5% paraformaldehyde/0.1% picric acid in HEPES buffer, pH 7.3) and the cells were fixed overnight. The fixative solution was removed, the pellet was washed in 0.1 M HEPES buffer (pH 7.3) twice for 5 minutes each, and the buffer was removed and replaced with a solution containing 1% osmium/1.5% potassium ferricyanide and incubated at room temperature in the dark for 30 minutes. The osmium-containing solution was removed and the pellet was washed 5 times in de-ionized water. The pellet was then incubated for 30 minutes at room temperature in aqueous 0.5% uranyl acetate. The pellet was dehydrated in a series of increasing alcohols and cleared with propylene oxide. The pellet was then embedded in epoxy resin and cured overnight at 55 C. Thin sections (80 nm) of the pellet were cut on a Leica Ultracut ultramicrotome using a Diatome diamond knife. The sections were placed on 200 mesh nickel coated grids and counterstained with uranyl acetate and lead citrate. Digital images through the meridian of individual cells (such that nucleoli were visible within the nucleus) were randomly taken throughout the section by a digital camera (AMT, Danvers, MA); this approach allowed for consistency of images taken between treatment groups. The area of the cells was determined using Image-Pro Plus software (Version 3.01, Media Cybernetics, Silver Springs, MD). Dense-core vesicles were identified based upon morphology (appropriately-sized vesicular structure with electron-dense core surrounded by halo of lucency) and were counted by hand by an individual blinded to treatment group. The density of dense-core vesicles/µm^2^ of cell cross-sectional area was calculated.

## Results

### shRNA-mediated knockdown of ZnT8 in INS-1 cells

**Figure 1:**
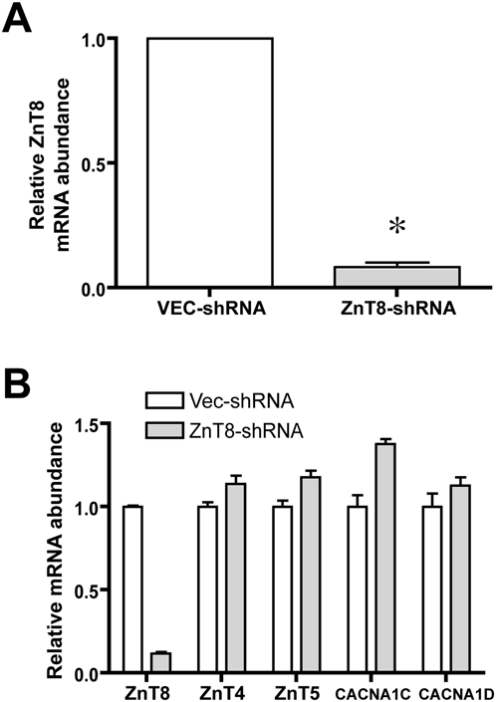
Effect of ZnT8-specific shRNA upon ZnT8 mRNA level in cultured INS-1 cells. A. Relative ZnT8 mRNA abundance as determined via real-time PCR in INS-1 cells were stably transfected with shRNA directed against rat ZnT8 (ZnT8-shRNA) or with shRNA vector alone (VEC-shRNA). Of note, the ZnT8-directed shRNA was comprised of two separate inserts (ZnT8-3 and ZnT8-4; see Table 1) cloned independently into expression vector and applied in combination. A. Stable cell lines were generated with these vectors on three separate occasions and the results from these three separate experiments are shown here (n = 3, with one determination per experiment). * denotes *p*<0.0001 relative to VEC-shRNA. B. Specificity of ZnT8 downregulation. RNA prepared from cells treated as in A were assessed via real-time PCR (n = 3) for the presence of ZnT8, for the presence of additional beta cell-expressed ZnT family members (ZnT4 and ZnT5), and for the presence of the alpha-1C and alpha-1D subunits of the beta cell-expressed L-type voltage-gated calcium channel (i.e., CACNA1C and CACNA1D gene products). For each mRNA, expression data were normalized to VEC-shRNA cells.

We adopted an shRNA approach to knockdown ZnT8 in INS-1 cells; cells were stably transfected with vectors coding for shRNA constructs (HuSH 29mers; OriGene, Rockville, MD). We initially screened a panel of four shRNAs (see Table 1). Two of these shRNAs (3 and 4) resulted in decreased expression of ZnT8 upon stable transfection in INS-1 cells (36% and 44% decrease in mRNA expression, respectively; n = 1). Thereafter, cells were transfected with a *combination* of vectors 3 and 4 to maximize effectiveness. The first set of stable transfectants was created by transfecting INS-1 cells with vectors 3 and 4 (in combination), or with the empty control plasmid. We adopted a pooled stable transfectant strategy as we have used previously to minimize the effect of integration site bias; however, because of potential selection bias in pooled stable transfectants, this entire stable transfection procedure was independently repeated twice more. In all, three complete *sets* of stable transfectants were generated and analyzed. Transfection with the combination of two ZnT8-directed shRNAs decreased ZnT8 mRNA expression by an average of 92±2% (p<0.0001; Figure 1; panel A). Reduction in expression was 88%, 93%, and 94% in the first, second, and third sets of stable transfectants, respectively. Of note, stable transfection of INS-1 cells with an irrelevant shRNA (directed against GFP) in a total of three separate transfectants exhibited no effect upon ZnT8 mRNA abundance (1.08±0.17, relative to Control; data not shown). As additional controls to assess for specificity of ZnT8 downregulation, the effect of ZnT8-directed shRNA upon abundance of other beta cell-expressed zinc transport proteins [34]–[36] and upon subunits comprising the L-type voltage-gated calcium channel was quantified via real-time PCR. Neither ZnT4 nor ZnT5 were down-regulated by the ZnT8-directed shRNA; similarly, the products of the CACNA1C and CACNA1D genes (coding for the α-1C and α-1D subunits of the beta cell-expressed L-type voltage-gated calcium channel, respectively) were not down-regulated by the treatment (Figure 1; panel B). There is at present no satisfactory antibody for detecting ZnT8 immunoreactivity. We tested one commercially available reagent; however, we were unable to detect ZnT8 expression in wild-type INS-1 cells or in lysates from pancreas (see Methods). Because we were unable to quantify the effect of our shRNA approach upon ZnT8 protein abundance, we resorted to a functional assay (see below).

### Zinquin as a reporter for intracellular zinc content

**Figure 2:**
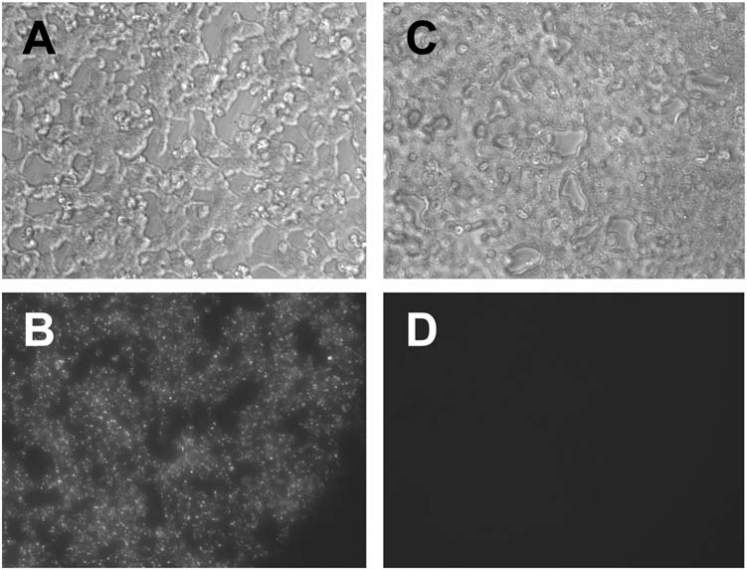
Zinquin staining in zinc- and nickel-treated INS-1 cell monolayers. A, C. Phase contrast view of untransfected INS-1 cells (original magnification: 200×) loaded with zinquin ethyl ester and treated for 3 h with zinc (75 µM ZnCl_2_; A) or nickel (75 µM NiCl_2_; C) and then loaded with zinquin as described in Methods. B, D. Epifluorescence views of the same fields depicted in A and C with emission monitored at 510 nm in the presence of excitation at 380 nm. Image pairs (A and C; B and D) were obtained using identical gain, binning, and exposure duration. Image pairs were combined into a single file (e.g., A+C, and B+D) prior to minor image processing in PhotoShop (Adobe) such that brightness and contrast were adjusted in parallel for the pair (see Methods for details). There is a lack of illumination in the lower right corner of panel B (and, imperceptibly, in panel D) as a consequence of the slight eccentricity of the epifluorescent illumination with respect to the brightfield illumination; viable cells were present in this region as evidenced in panel A but did not receive excitation.

Chimienti et al [8] and others have used the zinc-sensitive fluorescent dye, zinquin, to qualitatively assess changes in intracellular zinc concentration. We sought to test the feasibility of this method in the present context using fluorescent imaging of live cells in culture. INS-1 cells were loaded with zinquin ethyl ester (5 µM×30 min), exposed to either supplemental zinc or nickel as described above, and imaged under excitation at 380 nm and emission at 510 nm. There was negligible staining evident under control conditions (i.e., in the presence of only nominal levels of zinc; data not shown). There was robust staining in response to 3-h exposure of cells to supplemental zinc (75 µM; Figure 2; panel B), but negligible staining in response to 3-h exposure of cells to supplemental nickel (75 µM: Figure 2; panel D). Phase contrast views of the identical fields demonstrate intact cell morphology under both circumstances (Figure 2; panel A, C). The identical gain and exposure duration were used for the pair of A and C, and for the pair of B and D; post-processing of both image pairs was done strictly in parallel (see Methods). These data demonstrated the inhomogeneous staining characteristic of this assay (e.g., [8]).

### ZnT8-downregulated cells exhibit less zinquin-dependent fluorescence in the presence of exogenous zinc

**Figure 3:**
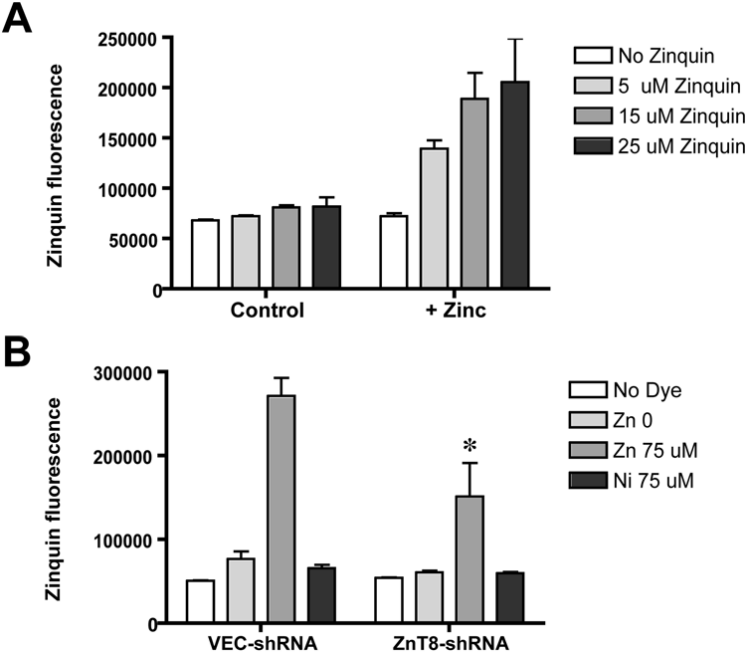
Effect of ZnT8 down-regulation upon intracellular zinc levels as assessed via zinquin fluorescence. In panel A, untransfected INS-1 cells in 96-well microtiter plates were treated for 3 h with control (nominally zinc-free) medium and Chelex resin-treated fetal bovine serum (10% vol∶vol), or the identical medium/serum combination supplemented with zinc (75 µM zinc sulfate). Cells were then washed, incubated for 30 min in HBSS with the indicated concentration of zinquin ester, and washed again. Each bar represents mean±SEM of twelve replicates; depicted figure is representative of two separate experiments. Fluorescence was measured with excitation at 370 nm and emission at 490 nm. Exogenous zinc markedly increases zinquin fluorescence at all tested concentrations of the dye. In panel B, this microtiter plate-based zinquin fluorescence assay was applied to stable cells expressing VEC-shRNA or ZnT8-shRNA, following incubation with control medium (Zn 0) or with medium supplemented with 75 µM Zn (Zn 75 µM) or Ni (negative control; Ni 75 µM). Fluorescence under control conditions in the absence of zinquin is also shown (No Dye). Each bar represents mean±SEM of three separate experiments (i.e., three sets of stable transfectants, each with twelve replicates). * denotes *p*<0.05 relative to the same experimental condition in VEC-shRNA stable transfectants. Down-regulation of ZnT8 resulted in less zinquin fluorescence in the absence (Zn 0) and presence (Zn 75 µM) of supplemental zinc; there was no effect upon fluorescence in the presence of nickel (Ni 75 µM) or in the absence of dye (No Dye).

The zinquin assay of intracellular zinc concentration was adapted for use in a medium-throughput microtiter-plate based assay. Untransfected INS-1 cells were treated with control medium or zinc-supplemented medium (+75 µM zinc sulfate) for three hours, and then sham-treated or loaded with varying concentrations of the zinc-sensitive dye, zinquin ethyl ester (5–25 µM×30 min). In the absence of zinquin, there was no difference in fluorescence (emission at 490 nm in response to excitation at 370 nm); however, the zinc-treated INS-1 cells exhibited more robust zinquin fluorescence than control-treated cells, and the effect was more pronounced at higher zinquin concentrations (Figure 3; panel A). Of note, the non-specific signal in the absence of zinquin dye – attributable to cell autofluorescence – serves as an internal control for cell plating density.

We next sought to compare zinquin fluorescence in vector-transfected INS-1 cells and in the ZnT8-downregulated INS-1 cells. In the vector-transfected (negative control) cells, zinquin fluorescence was much higher following exogenous zinc treatment (75 µM×3 h) than vehicle treatment (Figure 3; panel B). Cells were treated with an equivalent concentration of nickel ions as an additional negative control; there was only a very modest effect of exogenous nickel upon zinquin fluorescence. These data were consistent with the zinquin-dependent fluorescent signal reflecting intracellular zinc concentration. In ZnT8-downregulated INS-1 cells (ZnT8-shRNA), zinquin fluorescence was reduced relative to vector-transfected INS-1 cells. Even in the absence of supplemental zinc, zinquin-dependent fluorescence (i.e., fluorescence in the presence of zinquin, minus background fluorescence in the absence of zinquin) was reduced by 75% in the knock-down cells; although not reaching statistical significance (p = 0.16), such a phenomenon could reflect intracellular zinc retained from exposure prior to the 3-h incubation period. Following loading with 75 µM zinc, zinquin-dependent fluorescence was reduced by 56% in the ZnT8-down-regulated cells relative to the vector-transfected cells (p = 0.03). This suggested reduced intracellular zinc concentration in the ZnT8-downregulated cells. Of note, the background fluorescence in the absence of zinquin dye, reflective of cell plating density, was nearly identical in both groups (i.e., ∼6% higher in the ZnT8 knockdown cells, although this did not achieve statistical significance); this was unlikely to be a factor in the observed difference in zinc fluorescence. In aggregate, these data indicated that chronic ZnT8 downregulation influences intracellular zinc concentration and support an effect of ZnT8-directed shRNA upon ZnT8 protein expression. ZnT8 overexpression enhanced zinquin fluorescence [8], [18]; ZnT8-directed shRNA reduces it.

### ZnT8 knockdown cells exhibit less insulin immunoreactivity and reduced insulin secretion in response to glucose

**Figure 4:**
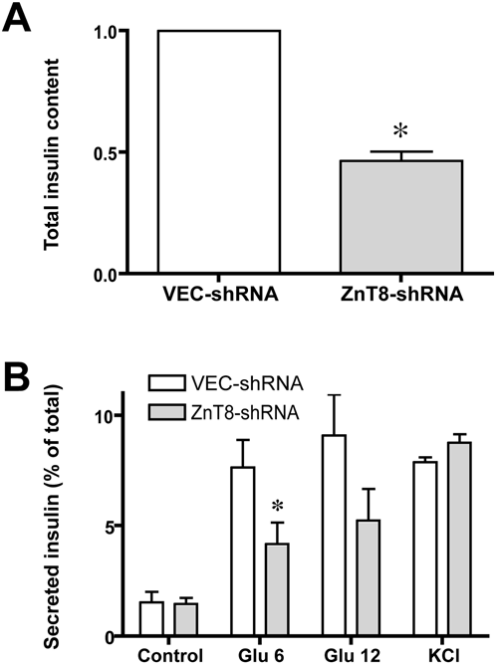
Effect of ZnT8 down-regulation upon intracellular insulin content and stimulated release of insulin. A. Intracellular insulin content (in ng/ml, and expressed relative to control) in INS-1 cells stably transfected with shRNA directed against vector (VEC-shRNA) or against rat ZnT8 (ZnT8-shRNA), as quantitated via immunoassay directed against rat insulin. Insulin content in the ZnT8-downregulated cells was 53.5% less (mean of data from three independently generated stable transfectants (n = 3), where each “n” represents 2–3 separate experiments per transfected cell line. Of note, total protein content per well for the VEC-shRNA and ZnT8-shRNA cells was equivalent (see Results). B. Insulin secretion (expressed as percent of total, i.e., 100*[secreted insulin/(intracellular insulin+secreted insulin)]) in VEC-shRNA and ZnT8-shRNA stable transfectants under control conditions, and in response to treatment with exogenous glucose (6 mM or 12 mM) or KCl (30 mM). “Control” condition denotes 3 mM glucose. Data are expressed as the mean±SEM for three independent stably transfected cell lines, where data for *each* transfection with a given cell line represents the mean of 3–5 individual experiments with an “n” of two replicates per experiment. * denotes p<0.05 with respect to vector-treated cells. The effect of glucose 6 mM achieved statistical significance (p = 0.04), whereas that of glucose 12 mM and KCl were suggestive of an effect (i.e., p = 0.09 and p = 0.09, respectively).

With this functional characterization in hand, we tested the effect of ZnT8 downregulation upon insulin secretion. ZnT8 downregulation reduced total intracellular insulin content by 53.5% relative to vector-transfected cells (Figure 4; panel A). Of note, there was no difference in total protein content per well between the vector-transfected and ZnT8-downregulated cells (541±102 vs. 610±96 µg/well, respectively; p = 0.2 for correlated samples), therefore this could not have accounted for the observed differences in insulin content. We next assessed insulin secretion in response to a glucose challenge (either 6 or 12 mM glucose supplementation, corresponding to 108 and 216 mg/dl, respectively), and in response to membrane depolarization with potassium chloride (30 mM). In vector-transfected cells, insulin secretion (expressed as percent of total insulin) was markedly increased by glucose treatment or KCl-mediated depolarization (Figure 4; panel B). Insulin secretion at baseline (Control) and in response to KCl-mediated depolarization was equivalent in vector-transfected and ZnT8-shRNA-transfected cell lines (Figure 4; panel B). Insulin secretion in response to 6 mM glucose was reduced by 45% in the ZnT8-downregulated cells (p = 0.04). The response to a higher glucose challenge (12 mM) was similarly decreased by 43% in the ZnT8-downregulated cells; however, this relationship was only suggestive and did not achieve statistical significance (p = 0.09). Although pooled data from all three sets of stable transfectants are shown in Figure 4 (panel B; where each set equals one “n”), this same pattern was evident in each of the three independent sets of stable transfectants (data not shown).

### ZnT8 down-regulation results in fewer dense core vesicles per unit cross-sectional area

**Figure 5:**
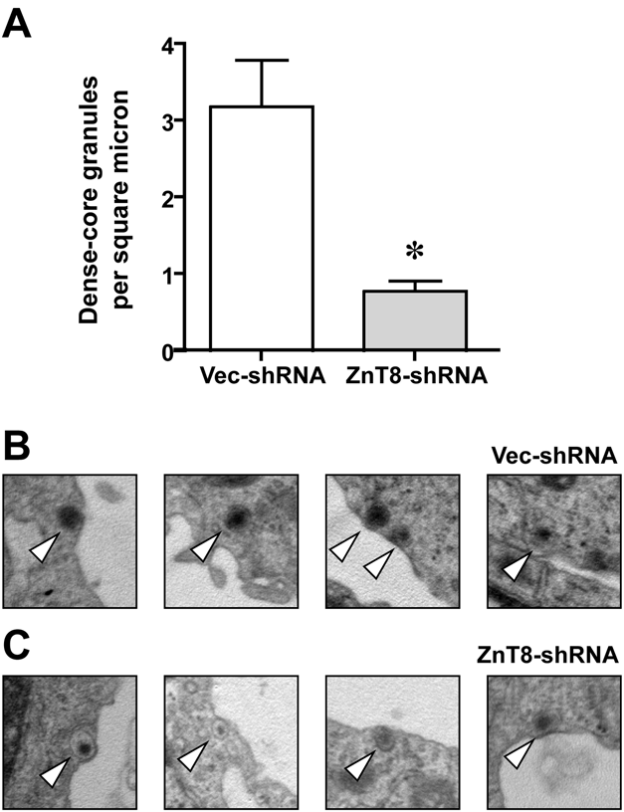
Effect of ZnT8 downregulation upon dense-core vesicle abundance. A. Abundance of ultrastructurally demonstrated dense-core vesicles in vector-transfected and ZnT8-shRNA-transfected INS-1 cells, expressed as the number of dense-core vesicles per square micron in cross-sectional images through the cell meridian. Mean±SEM of ten individual cells counted in blinded fashion is shown. Data were comparable for the first and third sets of stable transfectants; the second set was not tested. B and C depict representative vesicles (open arrowheads) in vector-shRNA (B) and ZnT8-shRNA (C) stable transfectants, respectively. Each image is 1 µm^2^ and the original magnification was 10,000×. Cells were approximately 6–10 µm in diameter and depicted vesicles are adjacent to the plasma membrane. * denotes p<0.05 with respect to vector-treated cells.

From an ultrastructural standpoint, dense-core vesicles are the site of insulin storage in pancreatic beta cells; elevated levels of circulating glucose cause the vesicles to fuse with the plasma membrane and secrete the insulin-rich contents (reviewed in: [1]). Therefore, we next tested for the presence of these vesicles via electron microscopy in the INS-1 stable transfectants. There were markedly fewer dense-core vesicles in the ZnT8-shRNA transfectants (0.8 vesicles vs. 3.2 vesicles per square micron of cell cross-sectional area; p<0.01; Figure 5; panel A). Of note, there were no gross differences in vesicle size, morphology, or subcellular distribution between groups (e.g., Figure 5; panels B,C).

## Discussion

These data indicate that down-regulation of ZnT8 expression in a cultured pancreatic beta-cell line results in reduced zinc fluorescence, insulin content, glucose-inducible insulin secretion, and dense-core vesicle number.

ZnT8 was identified after a search of a human expressed sequence tag database for motifs exhibiting similarity to any known human zinc transporter of the ZnT family [8]. ZnT8 expression at the mRNA level is restricted to pancreatic islets [8], [18], and a ZnT8-GFP fusion protein showed co-localization with insulin in INS-1 secretory vesicles [8]. Experiments based upon zinquin staining suggested that heterologous expression of ZnT8 in HeLa cells could confer enhanced cellular uptake of exogenous zinc [8]. Although no prior studies have examined the effect of ZnT8 downregulation, Chimienti and colleagues overexpressed ZnT8 in INS-1 cells [18]. In these studies, transfectants overexpressing the zinc transporter showed a marked increase in insulin secretion in response to hyperglycemic challenge. We hypothesized that downregulation of ZnT8 would decrease glucose responsiveness vis-à-vis insulin secretion; this effect was observed in the present studies.

The present data indicate that a reduction in the expression level of ZnT8 is associated with a marked decrease in intracellular insulin content in cultured rat insulinoma cells, and a reduction in the glucose-stimulated secretion of insulin. The reduction in this secretion – in absolute terms – is even greater than that implied by Figure 4; because ZnT8-downregulated cells express half as much insulin – on a “per mg of cell protein” basis – the absolute reduction in insulin secretion is even more dramatic. Nonetheless, secreted insulin is reduced even when assessed by the very conservative measure reported in Figure 4. Our findings, along with those of Chimienti et al wherein *over*expression of ZnT8 resulted in enhanced zinquin fluorescence and insulin secretion [8], [18], suggest a zinc-importing role for ZnT8; however, abundant data for other members of the ZnT family have led to the conclusion that ZnT proteins function primarily in zinc efflux from cells, or in its sequestration in intracellular vesicles (reviewed in: [37]). Whether this discrepancy reflects the unique functional properties of ZnT8 or an incomplete understanding of the role of ZnT8 in zinc trafficking will require additional investigation.

An understanding of the role of ZnT8 in the pathogenesis of diabetes is evolving rapidly. Shortly after the initial cloning of ZnT8, a polymorphism in the *SLC30A8* gene giving rise to a non-conservative amino acid substitution in the ZnT8 transporter was shown to be strongly associated with type 2 diabetes [3]; this association was subsequently replicated in numerous (e.g., [4]–[7]) but not all [38] genome-wide association analyses. The present data, in conjunction with the prior population-based studies suggesting a role for the diabetes-associated *SLC30A8* polymorphism in insulin secretion or action, raise the question of whether *SLC30A8* risk alleles directly impact ZnT8 expression level *in vivo*. As our current understanding evolves, such expression-level polymorphisms may supplant functional polymorphisms (i.e., those giving rise to loss or gain of function) as the primary drivers of inter-individual phenotypic variability [21]–[29]. Because the expression pattern of *SLC30A8* is so anatomically restricted, it will be challenging to assess for such variability in mRNA expression *in vivo*; pancreatic biopsies will likely not be undertaken for this purpose, and post-mortem examination may be confounded by the effects of years of diabetes. In addition to pancreas, ZnT8 was recently shown to be expressed in adipose tissue [39], which may prove more amenable to in vivo investigation. Although little is known about the mechanism through which the rs13266634 polymorphism in the *SLC30A8* gene confers susceptibility to diabetes, we speculate that reduced endogenous expression of the ZnT8 transporter constitutes one possible mechanism. However, irrespective of the mechanism of action of this one polymorphism, the present data suggest that expression-level polymorphisms have the potential to impact glucose homeostasis.

A number of caveats apply to the interpretion of the present data. First, although we reproducibly decreased ZnT8 expression at the mRNA level, we could not assess whether there had been a corresponding reduction in ZnT8 expression at the protein level. We infer downregulation of ZnT8 expression at the protein level based upon the specific inhibition in zinc-dependent zinquin fluorescence in the ZnT8-shRNA transfectants (e.g., Figure 3) – a phenomenon linked to ZnT8 abundance [18]. We infer specificity vis-à-vis ZnT8 because of the lack of effect upon abundance of other closely related beta cell-expressed ZnT family members (Figure 1B).

Second, measuring intracellular zinc can be problematic. The widely validated zinc-sensitive fluorophore, zinquin [8], [20], [40], was used in the present studies. We incorporated a number of important controls in support of our conclusion that intracellular zinc is reduced in INS-1 cells in response to ZnT8 downregulation. For example, zinquin fluorescence increased with zinquin concentration in a fashion steeply dependent upon supplemental zinc (Figure 3A). The issues of potential zinc toxicity and of zinquin specificity were addressed by the inclusion of the irrelevant metal ion, Ni^2+^, which failed to influence zinquin fluorescence (Figure 3B). In addition, extraneous sources of zinc were eliminated by using nominally zinc-free medium and by treating fetal bovine serum with the heavy metal-chelating resin Chelex as previously described (e.g., [31]).

Third, the effect of ZnT8 downregulation was specific to glucose-inducible insulin secretion and did not impact KCl-inducible insulin secretion. It is possible that the stimulus afforded by KCl depolarization, resulting in maximal recruitment of insulin secretory vesicles, was less susceptible to modulation; alternatively, the nature of the two stimuli – in contrast to the magnitude of the stimuli – may explain the difference. For example, ZnT8 may participate in the insulin secretory pathway upstream of membrane depolarization; however, there are no data to support this speculation at present. It is also important to note that the absolute amount of insulin secreted in response to both glucose and KCl was markedly less in the ZnT8-downregulated cells; however, the reduction in the insulin response to glucose was disproportionately low, relative to the reduction in total cellular insulin (i.e., data were expressed as percent of total cellular insulin and the ZnT8-downregulated cells expressed only approximately half as much insulin). In related fashion, KCl depolarization may be expected to result in more insulin secretion than glucose challenge. We saw substantial inter-assay variability and in some individual experiments, the effect of KCl exceeded that of glucose; however, the aggregated data showed similar responses in the control (vec-shRNA) stable transfectants.
